# Thromboembolism in the left main coronary artery of a patient with membranous nephropathy: a case report

**DOI:** 10.3389/fcvm.2026.1749262

**Published:** 2026-03-02

**Authors:** Baoguo Wang, Ming Lu, Hongjun Zhang, Chaoqun Huang, Weihua Zhang

**Affiliations:** Department of Cardiology, The First Hospital of Jilin University, Changchun, China

**Keywords:** acute myocardial infarction, hypercoagulability, left main coronary artery, membranous nephropathy, thrombosis

## Abstract

**Background:**

Membranous nephropathy (MN) is the most common cause of nephrotic syndrome in adults and a well-established risk factor for hypercoagulability. Although venous thromboembolism is frequently documented, arterial thrombosis—particularly acute thrombosis of the left main coronary artery (LMCA)—remains exceedingly rare.

**Case presentation:**

We report a fatal case of a 48-year-old male with a 10-year history of MN who presented with intermittent chest pain for 17 h and a marked elevation in troponin levels. Emergency coronary angiography revealed extensive thrombosis in the LMCA. Despite thrombus aspiration and stent implantation, the patient developed refractory cardiogenic shock and died.

**Conclusion:**

Acute LMCA thrombosis associated with MN is a rare occurrence. This case highlights that the hypercoagulable state linked to MN is not confined to venous thromboembolic events and may, in rare instances, lead to fatal arterial thrombosis.

## Case details

**Figure 1:**
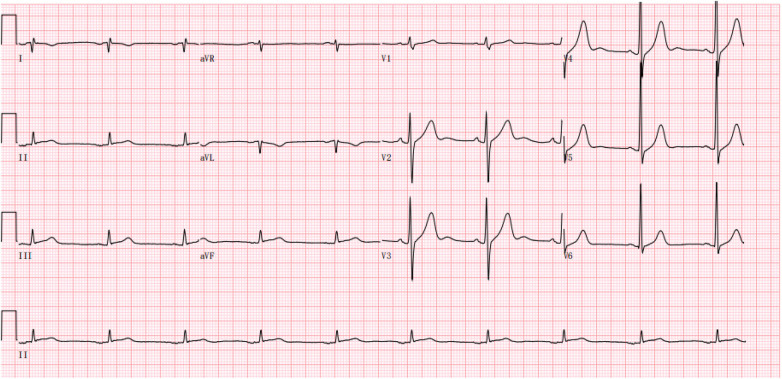
Electrocardiogram showed sinus bradycardia with a heart rate of 57 beats per minute.

**Figure 2:**
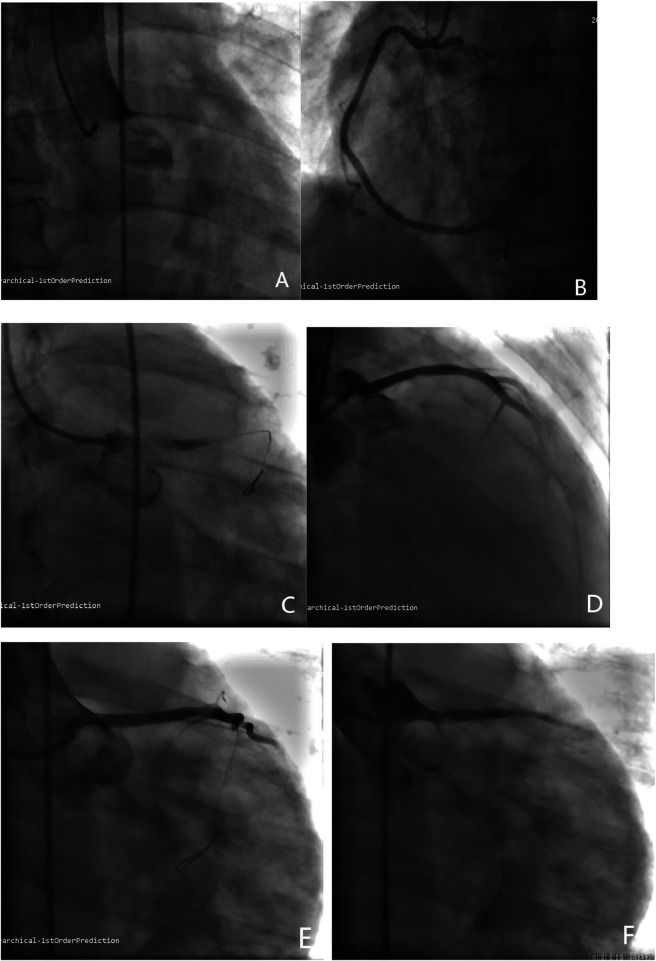
**(A)** Coronary angiography demonstrated complete occlusion of the LMCA with TIMI grade 0 flow. **(B)** Coronary angiography demonstrated no significant stenosis in the RCA with TIMI flow grade 3. **(C)** Coronary angiography demonstrated a large thrombus causing complete occlusion of the LMCA. **(D)** Coronary angiography demonstrated a 3.5*32 mm drug-eluting stent was deployed in the LM-LAD segment with no significant residual stenosis and restored antegrade flow with TIMI grade 2. **(E)** The LCX could not be visualized and antegrade flow was TIMI grade 0 on angiography. **(F)** Coronary angiography demonstrated persistent non-visualization of the LCX and no improvement in flow (TIMI grade 0) after dilatation with a balloon.

A 48-year-old Chinese male presented with a 17-hour history of intermittent, oppressive chest pain. The pain was accompanied by profuse sweating and five episodes of vomiting, leading to his admission to the cardiology intensive care unit. His medical history included MN, confirmed by renal biopsy at our hospital ten years ago (December 2, 2014), concurrently diagnosed with hyperlipidemia. He had been treated with glucocorticoids, tacrolimus (switched to cyclophosphamide after 1.5 years), and simvastatin for over three years. However, he subsequently discontinued this therapy and did not attend regular renal follow-up. He had no history of hypertension, diabetes mellitus or smoking. On admission, physical examination revealed the following vital signs: body temperature 36.3°C, pulse 57 beats per minute, and blood pressure 102/76 mmHg (1 mmHg = 0.133 kPa). Scattered crackles (rales) were audible in the lungs, while the lower limbs showed no edema. The patient was conscious but in evident acute distress. Laboratory examination: Serum troponin I 25.96 ng/mL (reference range 0–0.04 ng/mL), D-dimer 533.33 ng/mL (reference range 0–500 ng/mL), BNP 151.86 pg/mL (reference range 0–96 pg/mL). The blood routine test indicates an increase in white blood cell count and other relevant laboratory test results are shown in the Table 1. Additionally, the previous laboratory results of this patient during his follow-up at our hospital are presented in the Table 2. Electrocardiogram showed sinus bradycardia with a heart rate of 57 beats per minute (Figure 1). Emergency coronary angiography demonstrated complete occlusion of the LMCA with TIMI grade 0 flow (Figure 2A), while the right coronary artery (RCA) showed no significant stenosis and maintained TIMI grade 3 antegrade flow (Figure 2B). The primary diagnosis: Acute coronary syndrome, acute non-ST-segment elevation myocardial infarction, Killip grade II, membranous nephropathy.

**Table 1:** Laboratory results in this hospitalization.

Inspection item	Numerical value	Unit	Range of reference
troponin I	25.96	ng/mL	0–0.04
D-dimer	533.33	ng/mL	0–500
BNP	151.86	pg/mL	0–96
WBC	15.27	10^9/L	3.5–9.5
RBC	5.92	10^12/L	4.3–5.8
HGB	173	g/L	130–175
PLT	290	10^9/L	125–350
serum creatinine	90.1	umol/L	58–110
blood glucose	8.9	mmol/L	3.9–6.1
APTT	21.4	s	21–33
PT	10.4	s	9.0–13.0
FBG	2.97	g/L	1.8–4.0
PTA	129.8	%	80–120
serum potassium	4.17	mmol/L	3.50–5.30
Serum sodium	135.3	mmol/L	137.0–147.0
Serum calcium	2.04	mmol/L	2.10–2.55

BNP, brain natriuretic peptide; WBC, white blood cell; RBC, red blood cell; HGB, hemoglobin; PLT, Platelets; APTT, activated partial thromboplastin time; PT, prothrombin time; FBG, fibrinogen; PTA, prothrombin activity;.

**Table 2:** Comparison of laboratory results between the initial hospitalization and the last follow-up.

Inspection item	Results in 2014.12	Results in 2018.2	Unit	Range of reference
Albumin protein	22.0	42.8	g/L	40.00–55.00
TC	16.33	6.27	mmol/L	2.60–6.00
TG	3.30	1.37	mmol/L	0.48–1.80
LDL-C	11.73	4.42	mmol/L	2.07–3.10
24 h urine protein	6,487.0	686.7	mg/24h	0.00–200.00
24 h urine microalbumin	4,875.0	543.9	mg/24h	0.00–60.00

TC, total cholesterol; TG, triglyceride; LDL-C, low-density lipoprotein cholesterol.

**Figure 3:**
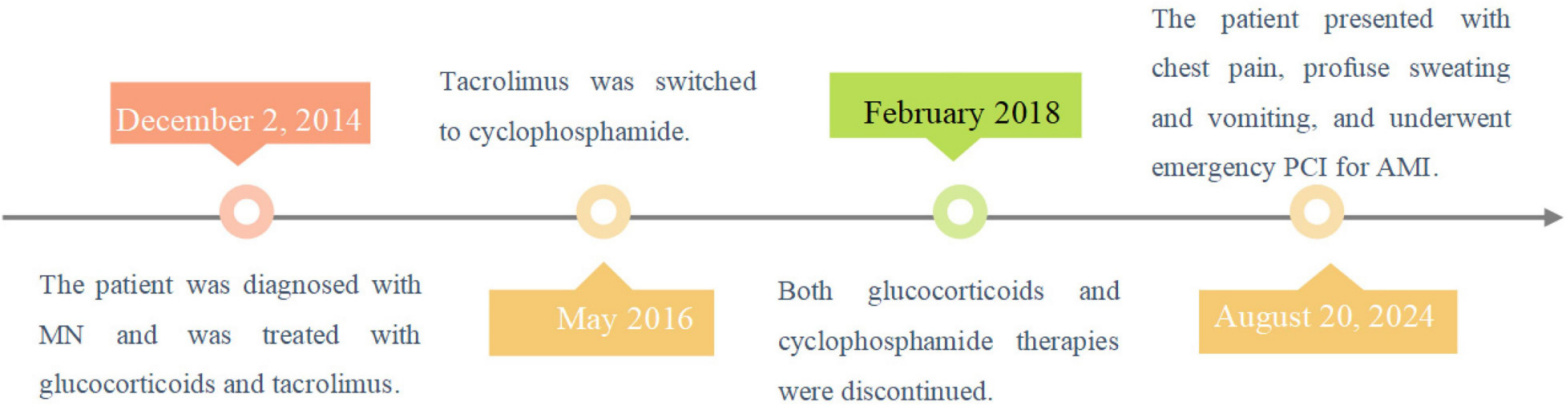
A timeline of all the patient's events.

Interventional procedure: At the start of the procedure, an 8,000-unit bolus of unfractionated heparin was administered intravenously for anticoagulation. The 6F EBU3.5 guiding catheter was successfully engaged. Subsequently, a SION guidewire was advanced into the distal segment of the left anterior descending (LAD) and coronary angiography demonstrated a large thrombus causing complete occlusion of the LMCA (Figure 2C). A thrombus aspiration procedure was then attempted by advancing the aspiration catheter over the guidewire; however, only a small amount of thrombotic material was successfully retrieved. Subsequently, a 2.5*15 mm pre-dilatation balloon was advanced over the guidewire and inflated in LM-LAD. A GP IIb/IIIa inhibitor (tirofiban, 1.5 mg) was then slowly infused through the guiding catheter directly into the culprit vessel. Finally, a 3.5*32 mm drug-eluting stent was deployed in the LM-LAD segment (Figure 2D). Post-procedural angiography demonstrated no significant residual stenosis and restored antegrade flow with TIMI grade 2. However, the left circumflex artery (LCX) could not be visualized and antegrade flow was TIMI grade 0 on angiography (Figure 2E). In an attempt to restore patency, a SION Blue guidewire was advanced into the distal LCX through the stent mesh, followed by dilatation with a 2.5 × 15 mm balloon. Repeat angiography, however, showed persistent non-visualization of the LCX and no improvement in flow (TIMI grade 0) (Figure 2F). Concurrently, the patient's hemodynamic status deteriorated precipitously. Blood pressure fell to 56/41 mmHg despite high-dose vasopressor support and fluoroscopy revealed severely diminished cardiac contractility. The electrocardiogram showed a ventricular rhythm with a rate of 50 beats per minute. Immediate cardiopulmonary resuscitation was initiated but was unsuccessful, and the patient died (Figure 3).

## Discussion and conclusion

MN is an autoimmune disease characterized by a thickening of the glomerular capillary walls due to immune complex deposition (1). And MN is the most common cause of nephrotic syndrome in adults, characterized by macroalbuminuria and hypoalbuminemia, resulting in edema and hypercholesterolemia (2–4). Up to one-third of the patients may reach spontaneous remission at any time during the course of follow-up (the majority within the first 2 years), one-third may persist with nephrotic syndrome, half of which may finally develop end-stage kidney disease (3). While MN predominately occurs between ages 40–75 years, it can manifest earlier (2). This 38-year-old patient presented with bilateral lower-extremity edema and significant proteinuria, leading to renal biopsy that confirmed MN. Following exclusion of diabetes, hepatitis, and multiple myeloma, primary MN was diagnosed.

The imbalance of promoters and inhibitors of the coagulation system leads to the thrombophilic state, which is more pronounced in MN than in other nephrotic disorders for as yet unknown reasons (1). Adults with MN develop thromboembolism at a rate of 8%–37%, usually during the first 2 years of presentation, with renal vein thrombosis occurring in 30% of these patients (1, 2, 5). Thrombotic complications in patients with MN occur more frequently in the venous than the arterial system, and coronary thromboses are particularly uncommon (6, 7). The hypercoagulable state in MN predisposes patients to venous thromboembolic events, including deep vein thrombosis, renal vein thrombosis, and pulmonary embolism. The risk of these complications is closely linked to the degree of hypoalbuminemia characteristic of the nephrotic syndrome (1, 7). In addition, hyperlipidaemia, especially an increase in LDL cholesterol, can result in acceleration of atherosclerosis and increase the risk of myocardial infarction or cerebrovascular accident (stroke) (1, 5, 8). Hypoproteinemia and hyperlipidemia can synergistically increase the risk of thrombosis. Systemic inflammation and endothelial dysfunction are recognized contributors to the elevated incidence of cardiovascular events in patients with MN. Concurrently, obesity constitutes an independent risk factor that further amplifies the cardiovascular risk profile in this patient population (9). A retrospective cohort study showed that absolute 10-year risk of cardiovascular events was 17.6% in adult MN patients (10). Another retrospective study showed that myocardial infarction and coronary artery disease had highest incidence in the MN group (7.2% and 14.5%, respectively) for hospitalized patients with nephrotic syndrome (8). In addition, a retrospective study of 1,800 patients with acute myocardial infarction (AMI) identified five individuals with concurrent MN, in whom the culprit lesions involved the RCA and LAD (6). However, acute LMCA thrombosis in the setting of MN rarely reported in the literature.

A comprehensive differential diagnosis was pursued. Antiphospholipid syndrome and inherited thrombophilia were unlikely given the negative rheumatologic/immunologic workup during the initial hospitalization and the absence of a family thrombotic history. Similarly, malignancy-related hypercoagulability was also rendered less likely by the negative tumor markers during the initial hospitalization. In addition, there was no significant stenosis in the RCA and no observable stenosis or plaque in the mid-LAD on angiography. Furthermore, the non-visualization of the LCX following stent implantation is consistent with the detachment and embolization of thrombus into that vessel. Therefore, given the patient's established history of MN and associated hypercoagulable state, along with the absence of angiographic evidence of plaque rupture, *in situ* thrombus formation is considered the most likely mechanism. Notably, a prior literature report describes a case of diffuse and refractory coronary thrombosis during percutaneous coronary intervention (PCI) in a patient with thrombotic predisposition (11), and the present case aligns with this phenomenon, underscoring the relevance of this mechanism and importance of intensified perioperative antithrombotic management for such patients.

Management of MN should aim to reduce proteinuria and control cardiovascular risk. This includes RAAS blockade, blood-pressure control (target ≤125/75 mmHg), a low-sodium (<2 g/day) and low-protein (0.8 g/kg/day) diet (2). Statin therapy also plays a clinically relevant role, despite the absence of formal guidelines for its use in nephrotic syndrome (5, 8). The 2021 Kidney Disease Improving Global Outcomes (KDIGO) guideline recommends assessing the risk of thrombotic events and bleeding complications in patients with MN according to the levels of serum albumin (12). Patients with MN need long-term monitoring, and patients with severe MN (proteinuria >10 g per 24 h and serum albumin <20 g/L) are at a high risk of thromboembolism and should be considered for prophylactic anticoagulation (2). A study pointed out that the prophylactic use of LMWH is the highest-rated evidence-based regimen in the prevention of thromboembolism in MN combined with hypercoagulable state (13). Antiplatelet drugs should also be administered on an individualize therapy in patients with MN (13). Immunosuppressive treatment in those at risk for kidney failure is recommended according to the 2021 KDIGO guidelines (12). This patient was treated with glucocorticoids, tacrolimus (switched to cyclophosphamide after 1.5 years) and simvastatin after the diagnosis of MN and he undergone regular renal follow-up for over three years. Despite achieving remission at last outpatient visit, the patient discontinued therapy and was lost to follow-up. The patient's LMCA thrombus is likely related to the ineffective control of MN, with an elevated D-dimer confirming a hypercoagulable state.

AMI with occlusion of an unprotected LCMA is a rare condition and 55% patients died within 24 h (14). The management of patients with acute thrombosis occlusion of the LMCA is challenging (15). Primary PCI is the standard of care for acute LMCA occlusion (16). However, despite emergency PCI and thrombus aspiration, the extensive thrombus burden and failure to achieve reperfusion in the LCX culminated in irreversible cardiogenic shock. This outcome highlights the limitations of mechanical intervention in the face of a systemic prothrombotic state. Intracoronary thrombolysis is complex in these patients due to increased risk of bleeding, but it may be beneficial in a life-threatening LMCA occlusion (17). In addition, mechanical support devices such as an intra-aortic balloon pump, Impella®, or extracorporeal membrane oxygenation may be necessary (14, 18). Unfortunately, due to the acute and rapidly deteriorating clinical condition, neither intracoronary thrombolysis nor mechanical support devices therapy was administered in this case. This case report is subject to several limitations. First, the urgency of clinical management during hospitalization limited the extent of renal evaluation and the thorough exclusion of alternative hypercoagulable disorders (e.g., antiphospholipid syndrome, inherited thrombophilia, and malignancy-related hypercoagulability). Thus, a potential contribution from these disorders cannot be entirely excluded. Second, the absence of echocardiographic evaluation means that a coronary embolism originating from an intracardiac source cannot be definitively ruled out. Third, as intravascular imaging (Intravascular Ultrasound or Optical Coherence Tomography) was not utilized during PCI, we lack definitive evidence regarding the presence of an underlying vulnerable or erosive atherosclerotic plaque beneath the thrombus.

In conclusion, we present a fatal case of acute LMCA thrombosis in a patient with MN. This report highlights that the hypercoagulable state linked to MN can lead to severe arterial thrombotic events. For cardiologists, this case underscores the importance of maintaining a high clinical suspicion for acute coronary syndromes in MN patients who present with chest pain, irrespective of traditional risk profiles. Furthermore, it calls for increased clinical vigilance and prompts consideration of the role of prophylactic anticoagulation in high-risk MN patients for nephrologists. Finally, for interventional cardiologists, this scenario emphasizes the necessity of being prepared to manage such catastrophic presentations, including the use of mechanical circulatory support devices when conventional interventions are insufficient.

## Data Availability

The original contributions presented in the study are included in the article/Supplementary Material, further inquiries can be directed to the corresponding author.
